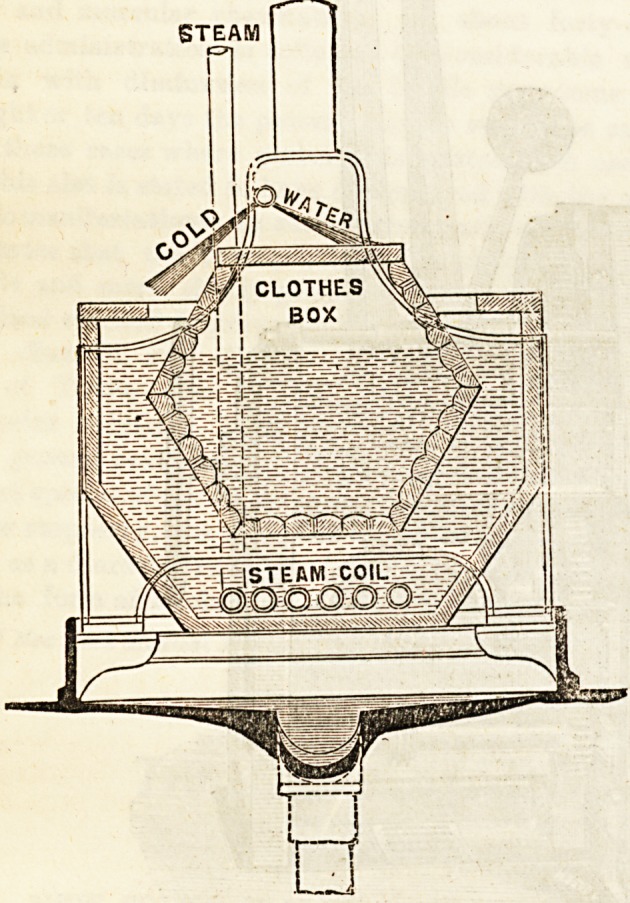# The Laundry

**Published:** 1892-09-10

**Authors:** 


### THE INSTITUTIONAL WORKSHOP.

### PRACTICAL DEPARTMENTS.

### I.—THE LAUNDRY.

We usually find a laundry attached to all institutions where the site is sufficiently spacious to allow of this convenience. Sometimes a whole block of the building is given up to it, but this arrangement is impracticable in towns where land is costly and scarce, and therefore the basement or the top storey has to be utilised for the purpose. In the latter case a flat roof probably serves for a summer drying ground.

We need hardly enlarge on the many advantages which attend "washing at home," as the familiar saying is, but briefly the arrangement is (1) economical, (2) convenient, and (3) sanitary.

Of the first, as regards institutions in general, there cannot be two opinions, for when the initial cost of the machinery is defrayed, the working expenses are wonderfully low. The engine, satisfactorily fed with the cheapest of fuel, not only furnishes steam power for the laundry, but also supplies the whole of the heating apparatus as well as all the hot water required in a large establishment. Secondly, the convenience is specially evident in hospitals and infirmaries where the quantity of linen required for the inmates' use is simply enormous, and can only be adequately supplied by the constant washing of the stock.

Thirdly, the sanitary advantages of having the laundry on the premises are as obvious as they are numerous. The sooner foul linen is cleansed, the better it is for everyone concerned, and naturally this immediate result cannot be otherwise attained. Comparatively little hand labour is necessitated by the earlier stages, as modern machinery proves itself equal to the varied demands made upon its resources.

Each institution displays some administrative individuality, but the general principles of the practical work are pretty much the same in all good laundries. The washing for the staff is always done separately, often in a special department, but at any rate quite distinct from that of the other inmates.

The linen used by typhoid patients has different treatment, and is generally of distinctive make or colour; it is disinfected, and washed in tanks set apart for the purpose.

The clothes from the obstetric wards are also treated by themselves during the various processes which they undergo. And there are always certain victims to diseases, of which it is not desirable to speak too definitely, both in workhouses and general, as well as special, hospitals, whose belongings have to be subjected to a most careful and isolated purification. It is only the experienced and responsible worker in institutions who can fully grasp the seriousness of all that appertains to conscientious laundry work, and it is well for anybody who is interested in the matter to study it thoroughly. It is easy to do this now-a-days, for there are numerous well-ordered establishments where the experience of several years enables the merits of all kinds of machines to be justly appraised, and we may add that personal experience has taught us that we can count on receiving courtesy and intelligent information whenever we take the trouble to seek for knowledge in institution laundries. In institutions other than sick asylums, everything goes to the laundry on specified days, but it is the custom to send them down each day in hospitals and infirmaries, and at all hours.

The nurse or maid closes the tightly-fitting door of "the shoot," after dispatching her cargo down it, with an immediate sense of transferred responsibility, but this channel of communication is but the first stage in the elaborate and rapid system which culminates in the airing closet. Sometimes a hoist or lift is used instead of "the shoot," but the design in all cases is the same, namely, the removal from the wards to the laundry of all condemned articles with the least possible delay. A duplicate list is kept of every thing sent down, and this is checked on the return of the clean linen, but it is not very surprising to find that the figures do not always agree. However, under careful management the losses ought to be very rare, and when small articles go astray into different departments they are generally promptly returned. The condition and correctness of the linen stores reflect credit or discredit at "inventory" times, and heads of departments take pride in earning a reputation for competent supervision of their stock.

A great deal of the success of the laundry work depends on the kinds of machines which are in use, and we may begin our practical hints by some allusions to different "washers." Many, excellent in themselves, and admirably suited for small establishments, are quite unfit to meet the demands made upon them by a great institution where the work is incessant and the quantity of articles very large.

Our illustration represents a steam-power washing machine which is very popular with the workers at the institutions where it has been in use for some time. We always attach value to the volunteered remarks of men and women employed in a laundry. They are generally to the point, and of practical assistance to anyone wishing to judge fairly of the relative merits of the machinery; and when the latter can be worked with no undue exertion on the part of the assistants, who bear at the same time testimony to details of the result, the verdict is a satisfactory conclusion to the opinion received from the manager. The "washer" here shown is in use at various asylums, such as the large one at Lancaster, at Durham, and Salford Workhouse and Sanatorium. In London, amongst other places, it is worked at Guy's Hospital, Stepney and Camberwell Workhouses and Infirmary, at the South Eastern Fever Hospital, and at Bermondsey Workhouse. Bermondsey, by the way, is an unsavoury neighbourhood; sensitive persons are known to draw up the windows of the carriage as their train passes through it, whilst a walk along the streets in the districts behind and beyond London Bridge station, convinces us that if disagreeables are "good for us," as some sects teach, we are for the moment recipients of an unfair share of these bounties. On penetrating to St. Olave's Union, and being courteously made free of the laundry, we are pleasantly surprised to find that we have not only left the local perfumes outside the building, but that they are replaced by no others. In fact, there is no smell whatever in any part of the place, the mechanical and automatic ventilations being admirably contrived and specially arranged by the makers of the laundry appliances, Messrs. Thomas and Taylor, of Stockport and Fonthill Road, Finsbury Park. The sectional drawing given here shows the clothes-box made of perforated copper (wood or metal) in which linen, however soiled, can be straightway placed, and need not be touched again by hand until it is perfectly cleansed. The outer casing contains the noiseless steam jet (steam coil) and also the water. The washing-box being full of perforations, allows the water to escape whilst retaining the clothes; and, if desired, a partially open tap allows a constant stream to flow through the machine whilst at work. The inside of the box is slightly corrugated, and this, together with the rotary action, turns the clothes in the water, whilst the motion of the box dashes them from end to end, and the two movements cause friction of the linen with itself and with the water, thus washing most effectually, rapidly, and gently, hard or soft, coarse or fine, materials.

*(To be continued.)*

## Figures and Tables

**Figure f1:**
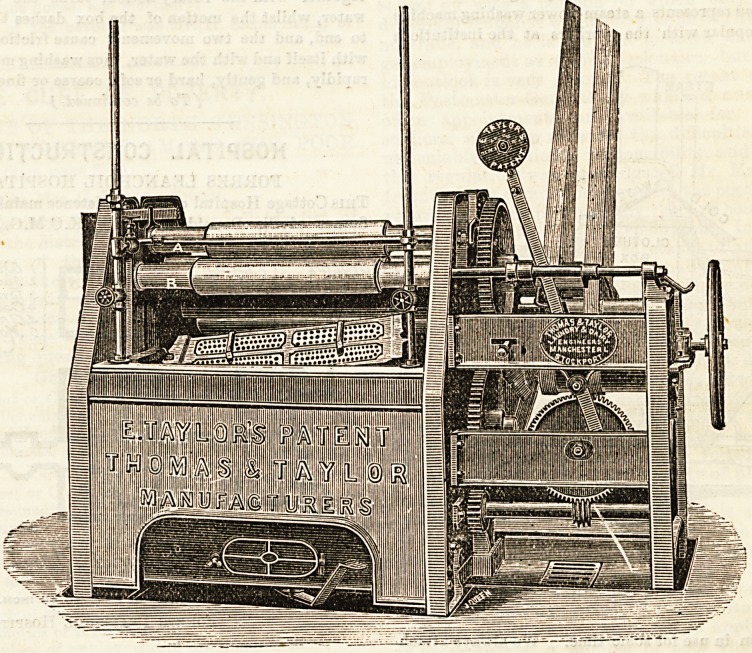


**Figure f2:**